# A New Silicon Dioxide-Coated MALDI-ToF Sample Plate for Peptide Analysis

**DOI:** 10.1155/2020/8597217

**Published:** 2020-06-30

**Authors:** He Huang, Lei Sun, Haiyang He, Tianbao Xia

**Affiliations:** ^1^Department of Critical Care Medicine, The 960th Hospital of PLA Joint Logistics Support Force, No. 25 Shifan Road, Tianqiao District, Jinan, Shandong 250031, China; ^2^Department of Stomatology, 306 Hospital of PLA, No. 9 Anxiang Beili Road, Chaoyang District, Beijing 100101, China; ^3^Department of Dermatology, 306 Hospital of PLA, No. 9 Anxiang Beili Road, Chaoyang District, Beijing 100101, China

## Abstract

In this report, we describe the development and testing of a new coated plate which improves the sensitivity and accuracy in matrix-assisted laser desorption/ionization time-of-flight mass spectrometry (MALDI-ToF-MS). The coated plate was covered with a thin layer of hydrophobic silicon dioxide, which enabled sample enrichment due to the water repellent nature of the silicon dioxide surface. Sensitivity and required laser strengths were tested using peptide standards, with the results that these coated plates required lower laser power and showed increased sensitivity than that of common plates. Accuracy was tested using bacteria, saliva, and serum samples. The coated plates showed significantly increased degrees of accuracy through their capacity to reduce mass shift. The importance and necessity of accuracy analysis in the assessment of new sample plates, which is rarely described in other papers, is also discussed.

## 1. Introduction

Since awarding of the 2002 Nobel Prize for the development of MALDI, a considerable amount of work has continued with MALDI-ToF development to enhance its capacity for the identification and structural analyses of biological macromolecules. These studies have incorporated efforts from several disciplines including physics, mathematics, chemistry, and materials science in an attempt to improve the pretreatment, methods, analysis, and data processing of the samples under investigation [1, 2]. Among research focused upon sampling methods, the development of sample plates represents a critical area of investigation not only for basic scientists but also for MALDI-ToF manufacturers. As a result, a considerable amount of research and patent applications have been directed toward new and improved sample plates, including disposable plates, chip plates, and coated plates. Teflon-coated plates as produced by Bruker and silicon dioxide-based chips with 4 nL 3-HPA by Sequenom company have both been shown to demonstrate greater degrees of sensitivity than that obtained with common plates [3, 4]. In addition to sensitivity, the accuracy of these plates requires verification to establish their effectiveness. For large-scale routine usage, like that involved with clinical diagnoses, neither Teflon layer plates nor prepared silicon dioxide chips are sufficiently cost-effective or easy to produce [5]. With increasing applications in the use of MALDI-ToF, the development of more effective and economical plates is sorely warranted.

## 2. Material and Methods

The plate used in this study was coated with a thin hydrophobic silicon dioxide layer as achieved using the vacuum deposition technique (Batch No.: 1307, Beijing ATY Co., Ltd). P_14_R, the ACTH Fragment 18-39, insulin standard, TFA, formic acid, acetonitrile, and HCCA were all purchased from Sigma-Aldrich. Serum samples were obtained from the Department of Clinical Biochemistry, and Pseudomonas aeruginosa samples are from the Department of Microbiology at the PLA General Hospital. Saliva samples were obtained from the Orthodontic Department at the Stomatology School of Peking University.

Serum and saliva samples were pretreated with weak cation exchange magnetic beads (WCX, Bruker Daltonik Inc.) [6] according to the instructions provided by the manufacturer. The Pseudomonas aeruginosa samples were harvested with use of a 1 *μ*L microloop from the medium, with the pellet then suspended in 300 *μ*L distilled water. The suspension was vortexed until the pellet was dissolved. Ethanol (900 *μ*L) was added to inactivate the microorganisms, followed by vortexing of the suspension. After centrifugation for 2 min at 14000 rpm at room temperature, the supernatant was removed and 50 *μ*L of 70% formic acid was added followed by 50 *μ*L of acetonitrile. After centrifugation for 10 min at 18000 rpm at room temperature, the supernatant was retained for MALDI-ToF analysis. The mass spectrum was collected using a linear MALDI-ToF instrument (LT2 plus, Scientific Analysis Instruments Co., Ltd, UK).

### 2.1. Laser Strength and Sensitivity

Peptide standards and matrices were prepared according to the following description. P_14_R, the ACTH Fragment 18-39, and insulin were diluted to 100 fmol/*μ*L, and 10 *μ*L from each prepared solution was combined. Excess HCCA was added in 50% ACN with 0.1% TFA; then, 0.5 *μ*L of the mixed standard solution was pipetted onto the sample wells of both the coated sample and common plates followed by an immediate addition of 0.5 *μ*L matrix before it dried. The solvent was then allowed to evaporate, the samples were dried at room temperature, and plates were transferred to the vacuum chamber of the mass spectrometer with the accumulation number set to 56. The spectrum was acquired using separate laser strengths of 110 and 90.

### 2.2. Serum Samples

Prepared serum samples (0.5 *μ*L) were pipetted onto the sample wells of the coated sample and common plates, with 0.5 *μ*L matrix immediately added before it dried. The solvent was then allowed to evaporate, and samples were dried for 5 minutes at room temperature. Plates were transferred to the vacuum chamber of the mass spectrometer with the accumulation number set to 200, laser strength to 130, and mass range set from 1000 to 10000 [7].

### 2.3. Saliva Samples

Prepared saliva samples (0.5 *μ*L) were pipetted onto the sample wells of coated sample and common plates, with 0.5 *μ*L matrix immediately added before it dried. The solvent was then allowed to evaporate, and samples were dried for 5 minutes at room temperature. Plates were transferred to the vacuum chamber of the mass spectrometer with the accumulation number set to 200, laser strength to 130, and mass range set from 1000 to 6000 [8].

### 2.4. Bacteria Samples

Prepared samples (0.5 *μ*L) were pipetted onto the sample wells of the coated sample and common plates, with 0.5 *μ*L matrix immediately added before it dried. The solvent was then allowed to evaporate, and samples were dried for 5 minutes at room temperature. Plates were transferred to the vacuum chamber of the mass spectrometer with the accumulation number set to 200, laser strength to 130, and mass range set from 1000 to 10000 for serum samples [7], 1000 to 6000 for saliva samples [8], and 1000 to 8000 for bacteria samples [9].

## 3. Results and Discussion

### 3.1. Laser Power and Sensitivity

Results obtained using different laser powers and plates are shown as follows: With the laser power set to 110, maximal peak intensity obtained from the coated plate was 6300 while that from the common plate was 750. With a laser power of 90, maximal peak intensity in the coated plate was 380, but no signals were observed in the common plate. The peak intensity of final spectra was accumulated and calculated from the peak intensity of each spectrum according to the shot quantity parameters used in the running method. The accumulation process can be stopped manually if an established peak intensity value or requirement is achieved in order to save experimental time and avoid overaccumulation [1].

The cocrystal in the common plate was small and dispersive, while that in the coated plate was larger and irregularly accumulated. The enriched effect of the hydrophobic silicon dioxide surface indirectly improves the sensitivity of the instrument by forming spots with higher sample concentrations on the plate [10]. As a result, the coated plate achieves a better spectrum by manually searching for the “sweet point.” This feature of the coated plate is particularly beneficial in samples where it is difficult to attain a good spectrum and is very much dependent on searching for a “sweet point.”

### 3.2. Saliva, Serum, and Bacteria Samples

**Figure 1:**
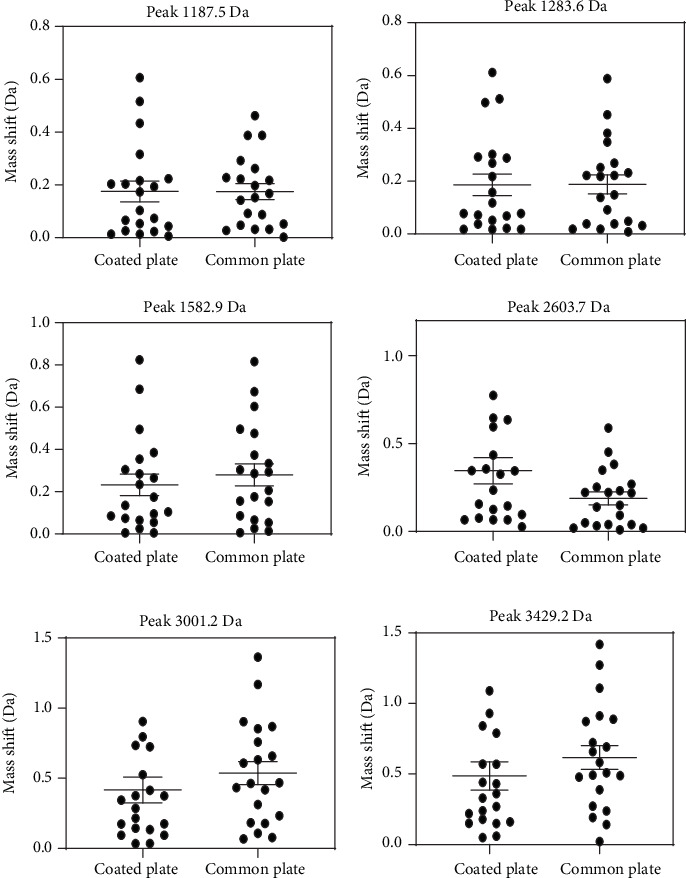
Comparisons of the mass shift levels from saliva samples as obtained on coated and common plates. At a peak of 1187.5 Da and 1286.3 Da, the mass shift level of the coated plate was slightly greater than that of the common plate. For the remaining four peaks, the means of mass shift levels of the coated plate were lower than those of the common plate. Values for this and Figures 2 and 3 are expressed as the mean ± standard error of the mean.

**Figure 2:**
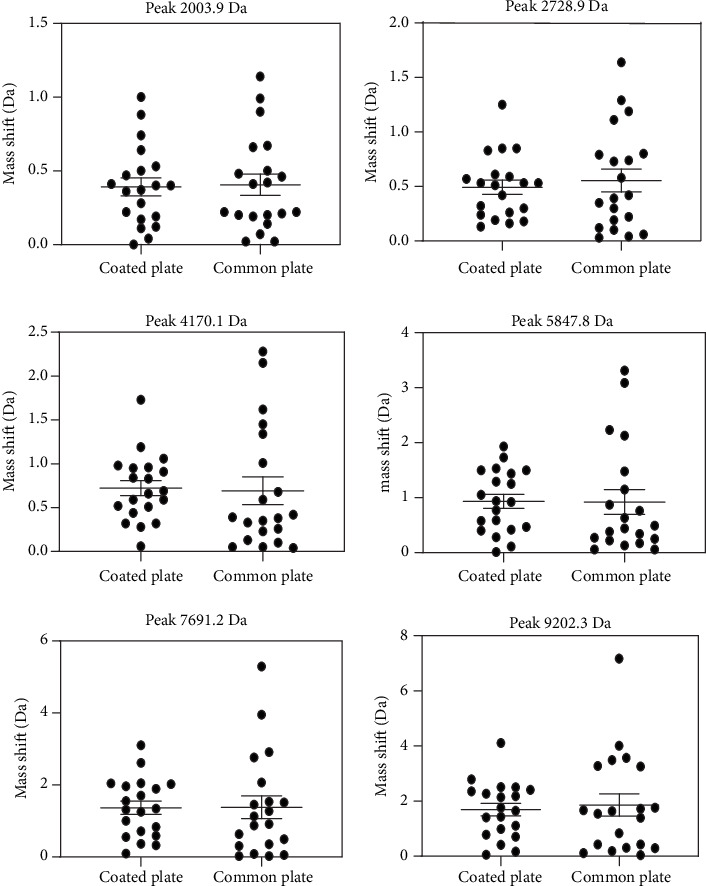
Comparisons of the mass shift levels from serum samples as obtained on coated and common plates. At a peak of 4170.1 Da and 5847.8 Da, the mass shift levels of the coated plate were greater than those of the common plate. For the remaining four peaks, the means of mass shift levels of the coated plate were lower than those of the common plate.

**Figure 3:**
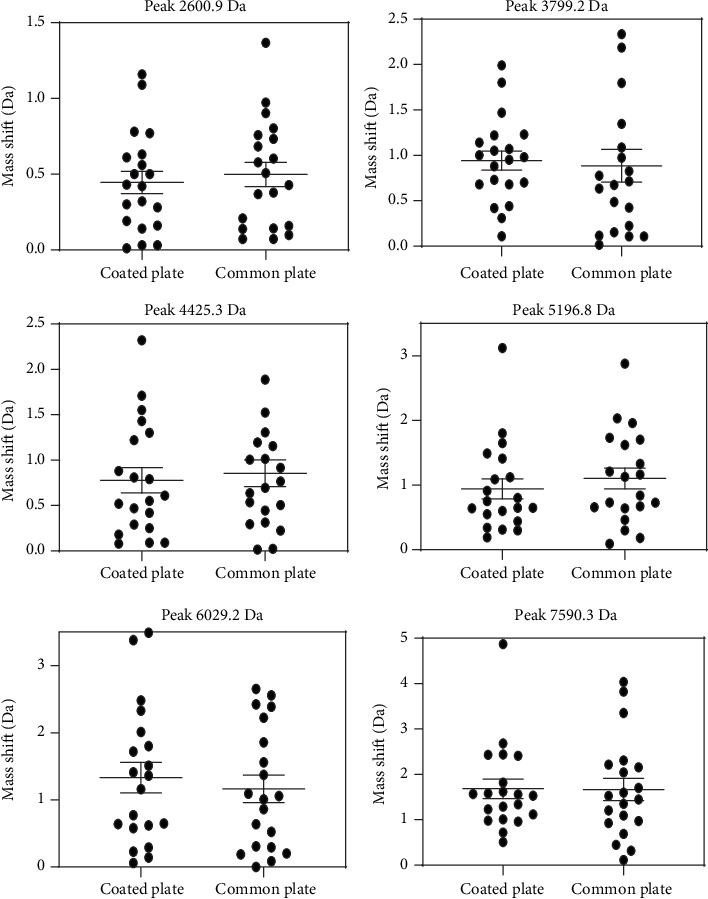
Comparisons of the mass shift levels from bacteria samples as obtained on coated and common plates. At a peak of 3799.2 Da and 6029.2 Da, the mass shift levels of the coated plate were greater than those of the common plate. For the remaining four peaks, the means of mass shift levels of the coated plate were lower than those of the common plate.

**Figure 4:**
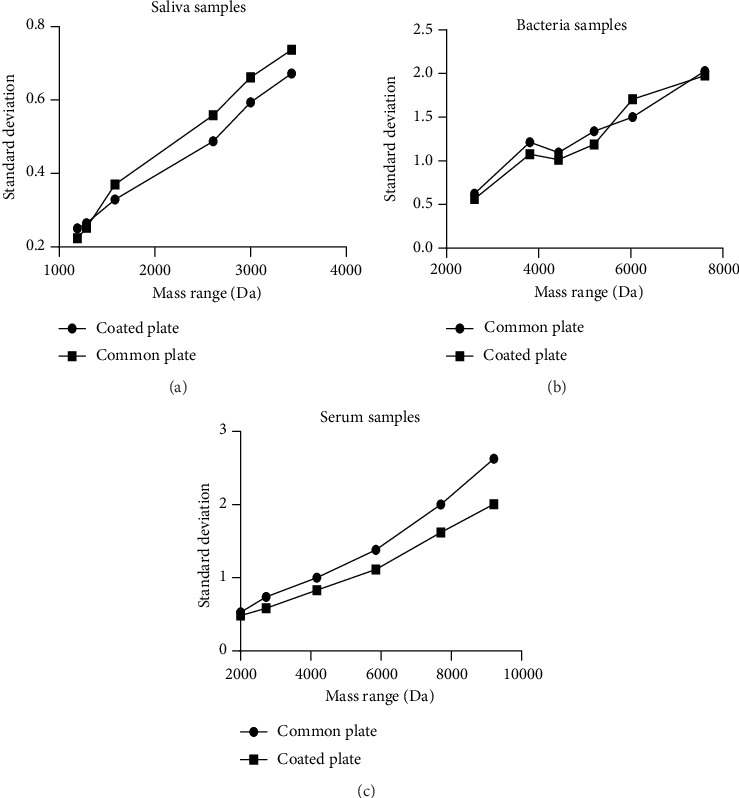
SD values for the 6 detected peak masses as obtained from the 20 samples: (a) saliva samples showing two peaks from the common plate with a lower SD than that of the coated plate; (b) bacteria samples showing only one peak from the common plate with a greater SD than that of the coated plate; (c) serum samples showing that all peaks from the coated plate were lower than those of the common plate.

For each of these samples, six peaks were chosen and used as the object of this study. These selected peaks included a sufficiently wide and equally distributed area of the entire mass range. The intensity of the chosen peaks should be of sufficient height to prevent them from being suppressed by either adjacent peaks or background noise. After comparing the peak shifts present in the three different sample types (Figures 1–3), our results reveal that the coated plate has the ability to reduce the level of mass shift for multiple chosen peaks and may also show this capacity for the majority of other peaks. Moreover, not only do the coated plates reduce the shift level but also decrease the distribution of the observed peaks. Although the SD results do not correspond with those of the mass shift results, results from the coated plate remain less distributed for multiple selected peaks as shown in Figure 4. One potential reason for the improvement in accuracy observed with coated plates may be due to an enhanced degree of homogeneity of cocrystals formed on the coated plate.

Previous studies involved with assessing sample plates mainly focus on cocrystal style or increases in the sensitivity of these plates [11], while accuracy analysis of plates is often ignored. This can occur, in part, because the application of external calibration can help to reduce the peak shift but does not eliminate this shift. In fact, one standard well is often employed to calibrate four, eight, or even more samples, making it difficult to properly calibrate all samples, especially those in the high mass region which show greater amounts of mass shift than that of the low mass region. Accordingly, the mass range of detected peaks is typically extended when using Mascot or similar searching engines, and plates that may result in more extreme mass shifts or distributions may be difficult to analyze and/or produce an erroneous result.

## 4. Conclusions

The coated plate demonstrated the ability to improve sensitivity and reduce the time expended on peak intensity accumulation. An additional notable advantage of the coated plate was the increased accuracy observed in the spectrum. The basis for this increased accuracy of coated plates remains unknown nor is it known why coated plates fail to improve the accuracy of all peaks. Additional issues to be addressed with regard to these coated plates include the performance of acid resistance, laser tolerance, and durability which will require testing in future work. While remaining cognizant of these issues, in this report, we demonstrate that, in addition to improved sensitivity and reductions in time expended on peak intensity accumulation, these coated plates show increased accuracy, and establishing the degree of accuracy represents a fundamental aspect in assessing the effectiveness of new plates or new coating technology.

## Data Availability

All data included in this study are available upon request by contact with the corresponding author.
